# Co-infections with *Plasmodium falciparum*, *Schistosoma mansoni *and intestinal helminths among schoolchildren in endemic areas of northwestern Tanzania

**DOI:** 10.1186/1756-3305-3-44

**Published:** 2010-05-19

**Authors:** Humphrey D Mazigo, Rebecca Waihenya, Nicholas JS Lwambo, Ladislaus L Mnyone, Aneth M Mahande, Jeremiah Seni, Maria Zinga, Anthony Kapesa, Eliningaya J Kweka, Stephen E Mshana, Jorg Heukelbach, Gerald M Mkoji

**Affiliations:** 1 Weill-Bugando University College of Health Sciences, P.O. Box 1464, Mwanza, Tanzania; 2 Department of Zoology, Jomo Kenyatta University of Agriculture and Technology, P.O. Box 62000-00200, Nairobi, Kenya; 3 National Institute for Medical Research, Mwanza Center, P.O. Box 1462 Mwanza, Tanzania; 4 Sokoine University of Agriculture, Pest Management Center, P.O. Box 3010 Morogoro, Tanzania; 5 Tropical Pesticides Research Institute, Division of Livestock and Human Disease vector control, P.O. Box 3024, Arusha, Tanzania; 6 Anton Breinl Centre for Tropical Medicine and Public Health; School of Public Health, Tropical Medicine and Rehabilitation Sciences, James Cook University, Townsville, Australia; 7 Institute of Tropical Medicine and Infectious Diseases, Jomo Kenyatta University of Agriculture and Technology, P.O. Box 62000-00200, Nairobi, Kenya

## Abstract

**Background:**

Malaria, schistosomiasis and intestinal helminth infections are causes of high morbidity in most tropical parts of the world. Even though these infections often co-exist, most studies focus on individual diseases. In the present study, we investigated the prevalence of *Plasmodium falciparum-*malaria, intestinal schistosomiasis, soil-transmitted helminth infections, and the respective co-infections, among schoolchildren in northwest Tanzania.

**Methods:**

A cross sectional study was conducted among schoolchildren living in villages located close to the shores of Lake Victoria. The Kato Katz technique was employed to screen faecal samples for *S. mansoni *and soil-transmitted helminth eggs. Giemsa stained thick and thin blood smears were analysed for the presence of malaria parasites.

**Results:**

Of the 400 children included in the study, 218 (54.5%) were infected with a single parasite species, 116 (29%) with two or more species, and 66 (16.5%) had no infection. The prevalences of *P. falciparum *and *S. mansoni *were 13.5% (95% CI, 10.2-16.8), and 64.3% (95% CI, 59.6-68.9) respectively. Prevalence of hookworm infection was 38% (95% CI, 33.2-42.8). *A. lumbricoides *and *T. trichiura *were not detected. Of the children 26.5% (95% CI, 21.9-30.6) that harbored two parasite species, combination of *S. mansoni *and hookworm co-infections was the most common (69%). Prevalence of *S. mansoni *- *P. falciparum *co-infections was 22.6% (95%CI, 15.3-31.3) and that of hookworm - *P. falciparum *co-infections 5.7% (95%CI, 2.6-12.8). Prevalence of co-infection of *P. falciparum*, *S. mansoni *and hookworm was 2.8% (95%CI, 1.15-4.4).

**Conclusion:**

Multiple parasitic infections are common among schoolchildren in rural northwest Tanzania. These findings can be used for the design and implementation of sound intervention strategies to mitigate morbidity and co-morbidity.

## Background

Parasitic infections present a major cause of disease and morbidity in Africa [1]. Among these diseases, *Plasmodium falciparum *inflicts the largest burden [2,3]. It is estimated that 40% of the world's population is at risk of malaria, and about 90% of the infected populations with malaria live in sub-Saharan Africa [4]. In Tanzania, over 95% of the 38.7 million people are at risk for malaria; the disease contributes to 39% - 48% of all outpatients [5].

Schistosomiasis is endemic in 76 countries worldwide and besides malaria is the second important parasitic disease for public health [6,7]. Of the 662 million people infected worldwide, 85% are from Africa [8]. In Tanzania, schistosomiasis is highly endemic and its prevalence varies from one region to another, with a prevalence of up to 80% in highly endemic areas [9].

The World Health Organization estimates that more than one billion people are chronically infected with soil-transmitted helminths [10]. Hookworms are estimated to affect 1300 million people [11].

Due to the fact that *P. falciparum *malaria and helminth infections are endemic throughout Tanzania, the affected populations often endure infections with a number of different species [12], and individuals are commonly co-infected with combinations of helminths and malaria parasites [13]. Such infections may have considerable health consequences, leading to more severe clinical symptoms and pathology than for infection with single parasite species. Interaction of malaria and helminth infections increase the severity of anaemia and organomegaly observed in schoolchildren and thus may potentially create a great challenge for disease control in the tropics. There is also evidence that co-infection with multiple parasites may alter the immune responses [14].

Overlap of schistosomes, soil-transmitted helminth and *P. falciparum *malaria depends on the conditions that favour multiple parasitic species survival and transmission [15]. These conditions include poverty, environmental contamination, water bodies and lack of effective preventive measures [15]. Polyparasitism of *P. falciparum *malaria, schistosomiasis and soil-transmitted helminth have been reported from various epidemiological settings in Africa [12,16,17], yet most parasitic diseases are still studied individually and data on prevalence, morbidity and mortality associated with multiple parasitic infections in Tanzania are limited.

This study was therefore conducted to determine the prevalence of *P. falciparum *malaria, *S. mansoni *and soil-transmitted helminth, and co-infections, among schoolchildren living in villages surrounding the Lake Victoria shore. Understanding the extent of polyparasitism in high-risk groups will serve as a guide in developing sound interventions strategies to reduce the burden of these diseases and co-morbidity.

## Materials and methods

### Study area and population

Nyamatongo ward is located in Sengerema district in the Mwanza region, northwest Tanzania. The ward is located on the eastern side of the district and boarders Lake Victoria to the east. It has a population of about 21,000. The study area is located at 1140 m altitude above sea level, and the inland area is covered by seasonal rivers and stream flowing down to the lake. The area is characterized by a tropical climate, with an average annual temperature of 26.5°C, and experiences long rainy seasons between January and May. Annual average rainfall is 1065 mm. The majority of inhabitants are involved in rural subsistence farming, fishing and livestock keeping.

The study was carried out between March - May 2009 among 400 schoolchildren from four primary schools, located 500 m-1500 m from the shore of Lake Victoria in Sengerema district, northwest Tanzania. The inclusion criteria for the study were: (1) children aged 8-16 years; (2) parents or guardians gave written informed consent; (3) in addition to the written consent from caretakers, children also were supposed to agree and provide informed assent; (4) children lived in the study area.

### Study design

A cross-sectional study was undertaken to determine the prevalence of *P. falciparum *malaria, *S. mansoni *and soil-transmitted helminth and any co-infections of *P. falciparum*, and intestinal helminths among schoolchildren. A two-step random sampling method was used to select children participating in the study. First, four schools of Nyamatongo ward were selected randomly. Second, using the class attendance registers, each child was given a number. Selection of the study participants was achieved by using tables of random numbers. Demographic data including age, gender and school/village where participants studied/lived were recorded by a structured questionnaire.

### Parasitological examination of *S. mansoni *and soil-transmitted helminths

The day before parasitological screening commenced, schoolchildren were issued with plastic containers and instructed by a research team (HDM and NJSL) on how to collect a portion of their morning stool samples the next day. Stool containers were then collected at the school and labeled with identification numbers. These samples were brought to the field laboratory on the same day, and duplicate Kato-Katz cellophane thick smears was prepared from each specimen [18,19]. As a quality control measure, 10% of randomly selected smears were re-examined by a third experienced parasitologist who was blinded of the previous results. The mean number of eggs from each Kato Katz thick smear were multiplied by 24 in order to express infection intensities as the number of eggs per gram of faeces [18].

### Parasitological examination of malaria parasites

A finger prick blood sample was collected after cleaning the finger surface using a sterile cotton wool soaked in methylated spirit. Two thick and one thin smears were prepared and stained with 10% Giemsa (Sigma, Aldrich, Nairobi) in phosphate buffer [18]. Species specific and parasite densities were estimated under a light microscope at high magnification by counting the number of parasites per 200 white blood cells (WBC). If < 10 parasites were found the reading was continued up to 500 WBC. The presence of either ring forms or gametocytes was conclusive diagnosis of *P. falciparum*. The counts of *P. falciparum *were converted to the number of parasite per μl of blood, assuming as standard a WBC count of 8000/μl [20].

### Treatment

Children found positive for hookworm and *S. mansoni *infections were treated with mebendazole 500 mg and praziquantel 40 mg/kg [21]. Sweet potatoes and porridge were given before tablets in order to reduce the nauseating effects of praziquantel. Children positive for malaria parasites and body temperature above 37°C were treated with combination therapy of lumefantrine and artemether in collaboration with nearby health centers within the village.

### Ethical considerations

The study was approved by the Institutional Review Board (Research and Publication Committee, certificate no. BREC/001/03/2009) of the Weill-Bugando University College Health Sciences and Bugando Medical Centre, Mwanza, Tanzania.

Prior to conducting the study, meetings were held with parents or guardians, teachers and community leaders to explain the aims and procedures to be used to collect data. Informed written consents were obtained from children's parents or guardians and in addition assent was subsequently obtained from children.

### Data analysis

Data were entered into Microsoft Excel 2003 spreadsheets (Microsoft Corp., Redmond, WA, USA), checked for entry errors and analysed using SPSS for Windows version 11.5 (SPSS Inc., Chicago, IL, USA). Intensity of helminth infection was expressed as arithmetic mean from two Kato Katz thick smears [18]. Infection intensity of *S. mansoni *was classified into four groups according to WHO criteria: (1) light infection (1-100 eggs/g faeces (epg), (2) moderate infections (101-400 epg), (3) heavy infections (401-1000 epg) and (4) very heavy infections (> 1000 epg). For hookworms, infections were classified as follows: (1) light < 1000 epg; (2) moderate infection (1000-3999 epg) and (3) heavy infection (> 4000 epg) [20].

*P. falciparum *was specified on the basis of Giemsa stained blood smear examination, and infection intensities were classified into four groups: 1-50, 51-500, 501-5000 and > 5000 parasites/μl of blood [22].

The prevalence of single and multiple species of parasites were assessed and classified by gender and three age groups (≤ 10, 11 - 13 and 14-16 years). Chi-squared (X^2^) test was used to assess whether single and double or triple parasitic infections were associated with sex and age. Logistic regression analysis was applied to investigate whether gender and age were significantly associated with *S. mansoni, P. falciparum *and hookworms. A model of with *S. mansoni *infected children defined as cases incorporated age and sex. The adjusted odds ratios, including 95% confidence interval and *P*-values were calculated. Similar procedures were repeated for hookworm and *P. falciparum *infections.

## Results

A total of 400 schoolchildren aged 8-16 years were included. The age and sex distribution of study participants is shown in Table 1.

**Table 1:** Study population in Nyamatongo ward, stratified by age and sex.

Age (years)	Males	%	Females	%	Total
≤ 10	33	16.8	50	24.5	83
11 - 13	104	53.1	106	52	210
14 - 16	59	30.1	48	23.5	107
Total	196	100	204	100	400

In total, 218/400 (54.5%, 95% CI: 49.6-59.4) harboured at least one parasitic infection and 66/400 (16.5%, 95%CI 12.9-20.1) had none of the investigated parasitic infections.

The prevalence of *S. mansoni *was 64.3% (95% CI: 59.6-68.9) with no significant difference among males and females (Table 2). The majority of the children infected with *S. mansoni *(35.4%, 95%CI: 30.7-40.1) had light infection (1-100 epg).

**Table 2:** Overall prevalence of parasitic diseases investigated, stratified by sex, among 400 schoolchildren in Nyamatongo ward, northwest Tanzania.

		Prevalence of infection (%)			
	Overall N (%)	95% confidence interval	Females (n = 204) N (%)	Males (n = 196) N (%)	P-value (males vs. females)
**Schistosomes**					
*Schistosoma mansoni*	257	59.9-68.8	135	122	P = 0.31
	(64.3%)		(66.18%)	(62.24%)	
**Soil-transmitted helminths**					
Hookworm	152	33.7-42.8	78 (38.23%)	74 (37.8%)	P = 0.70
	(38.5%)				
**Plasmodia**					
*Plasmodium falciparum*	54 (13.5%)	10.2-16.9	34 (16.7%)	20 (10.2%)	P = 0.26
*Plasmodium ovale*	3 (0.8%)	0-10.9	2 (0.98%)	1 (0.51%)	
**At least one parasitic**	218	46.6-59.4	110	108	P = 0.39
**infection**	(54.5%)		(53.92%)	(55.10%)	
**Two or more parasitic**	116 (29%)	24.6-33.4	61 (29.90%)	55 (47.41%)	
**infections**					

The overall hookworm prevalence was 38.5% (95% CI: 33.2-42.8) with no significant differences between males and females (Table 2). Interestingly, prevalence of hookworms was observed to increase with age reaching maximum at 44.8% (95% CI: 40-49.8) in the 14 -16 years age groups. About 32% (95% CI: 27.2-36.3) of the children had light hookworm infections with only 1% of the children harbouring heavy infections. Infections with other intestinal helminths such as *T. trichiura *or *A. lumbricoides *were not detected. Table 2 displays the overall prevalences and prevalences stratified by sex of the parasitic diseases identified in the study population.

Logistic regression analysis revealed significant associations between *S. mansoni *and hookworm infections with the age group (14-16 years), *S. mansoni *(adjusted OR = 0.47, *P *< 0.05, 95% CI: 0.25-0.87) and hookworm infection (adjusted OR = 1.85, *P *< 0.002, 95% CI: 1.00-3.4), as compared to the ≥ 10 year age group. Infection with *S. mansoni *(adjusted OR = 0.84, 95% CI: 0.55-1.26, *P *= 0.41) as well as with hookworms (adjusted OR = 0.98, 95% CI: 0.65-1.46, P = 0.92) was independent of gender.

Out of the 400 children tested for malaria parasites, 57 (14.3%, 95% CI: 10.8-17.7) were positive, with a prevalence of *P. falciparum *of 13.5% (Table 2). Three children (0.8%, 3/400) were found to harbour *P. ovale*. None of the children harboured *P. vivax *or *P. malariae*. Malaria prevalence was highest in children aged 11-13 years at 15.3% (n = 30; 95% CI: 11.7-18.7) and females had higher prevalence of asymptomatic malaria (16.6%) (Table 3). About 65% of the children with *P. falciparum *infection had asymptomatic parasitaemia with 501-5000 parasites/μL of blood (Table 3). However, no significant association was observed between age groups and *P. falciparum *infections (*P *= 0.73). Co-infection with more than one parasite species was common. The overall prevalence of at least two parasite species in study participants was 26.5% (n = 106), 95% CI, 22.2-30.8) (Table 2). About 69% (73/106, 95% CI: 64.4-73.4) of the children with dual infections were co-infected with hookworms and *S. mansoni *(Table 4). Other dual infections observed were between *S. mansoni *+ *P. falciparum *(22.6%, 24/106), hookworm + *P. falciparum *(5.7%, 6/106). The prevalence of dual infections did not differ significantly by gender (*P *= 0.068). The age group 14-16 years exhibited high prevalence of dual infection (Table 4). Triple infections were only observed in 2.8% (n = 11/400; 95% CI, 2.3-3.2) of the samples examined and these were for *S. mansoni *+ hookworm + *P. falciparum*. Triple infections were most common in children in the age group 11-13 years and accounted for 72.7% of the cases (n = 8/11). Female children had the highest prevalence of triple infections (64%, 7/11).

**Table 3:** Infection intensity *of P. falciparum *infections, stratified by sex and age (n = 397, children infected with *P. ovale *were excluded).

	1-50 n (%)	51-500 n (%)	501-5000 n (%)	> 5000 n (%)	*P*-value (males vs. females)
**Sex**					
Male	0	5 (25%)	13 (65%)	2 (10%)	
Female	1 (2.94%)	10 (29.41%)	22 (64.71%)	1 (2.94%)	P = 0.26
**Age (years)**					
≤ 10	0	2 (22.22%	7 (77.78%)	0	
11 - 13	1 (3.13%)	7 (21.88%)	22 (68.75%)	2 (6.25%)	
14 - 16	0	6 (46.15%)	6 (46.15%)	1 (7.69%)	P = 0.73
**Total**	1	15	35	3	

**Table 4:** Prevalence of double and triple parasitic infections, stratified by age and sex among 400 primary schoolchildren in the Nyamatongo ward.

	Prevalence of double and triple infections				
Variable		*S. mansoni +* *P. falciparum*	*S. mansoni +* Hookworm	*P. falciparum +* hookworm	*P. falciparum *+ *S. mansoni *+ hookworm
**Sex**	N	N (%)	N (%)	N (%)	N (%)
Female	204	14 (6.9%)	33 (16.2%)	5 (2.5%)	7 (3.4%)
Male	196	10 (5.1%)	40 (20.4%)	1(0.5%)	4 (2.1%)
					
**Age (years)**					
≤ 10	83	4 (4.8%)	16 (19.3%)	0	2 (2.4%)
11 - 13	210	14 (6.7%)	33 (15.7%)	3(1.4%)	8 (3.8%)
14 - 16	107	24 (17.1%)	73 (57.4%)	6(1.42%)	1 (0.9%)
All age groups	400	42	122	9	11

## Discussion

The findings from the current study confirm that Nyamatongo ward in northwest Tanzania is highly endemic for intestinal schistosomiasis and hookworm infections. *S. mansoni *was the most prevalent parasitic disease identified with almost 2/3 of the schoolchildren infected. Prevalence of malaria was relatively low. Parasitic co-infections were common. Ascariasis and trichuriasis were absent.

Earlier studies conducted on other localities within the lake basin showed that both schistosomiasis and hookworm infections are common in the area [9,23]. However, prevalences in earlier studies were much lower (10.9%- *S. mansoni*) than in the present study probably due to focal exposure to *S. mansoni *by the population living in endemic areas as reported previously [24,25]. Infection intensities for *S. mansoni *in children were light to moderate, and only 7.5% of the children had heavy infections. These findings support previous observations that only few individuals in an endemic community excrete large numbers of eggs and that *S. mansoni *infections starts at early ages [26]. In the current study, children < 13 years recorded highest infection rates with *S. mansoni*. Similar observations have been made for other areas [27]. On the other hand, the prevalence of *S. mansoni *was observed to decrease with increase in age of the children, which has also been reported in previous reports from Cote d'Ivoire [27]. The decrease in prevalence of *S. mansoni *appears to coincide with the onset of puberty [25]. The prevalence of hookworm infections observed in the current study area (38%) was similar to Magu district (37%) within northwest Tanzania [9] but slightly lower than reported from Western Kenya (42.5%) [25] and Brazil [28].

Our data confirm that among soil-transmitted helminths, hookworm infection is the major public health problem within the Lake basin [9,25]. A positive association between older age group (14-16 years) and hookworm infection was observed. A similar observation was made in locality in rural Côte d'Ivoire [27]. Hookworms were more widespread as compared to other soil-transmitted helminth infections, namely *A. lumbricoides *and *T. trichiura*, which occur in various ecological settings of sub-Saharan African [29]. Interestingly, earlier studies in Magu district, northwest Tanzania, reported a prevalence of ascariasis of < 1% [9] and a report by Handzel *et al*. [25] in Kenya's Nyanza province reported a prevalence of 22.9% and 17.9% for *A. lumbricoides *and *T. trichuris*. Variability in endemicity or prevalence of these infections, low sensitivity of the diagnostic method, the use of single stool sample, environmental contamination and inability of the helminth eggs to withstand high temperature could partly explain the observed difference. The prevalence of *P. falciparum *malaria in Nyamatongo ward was relatively low (13.5%) in spite of the locality being within a malaria holoendemic area [30]. Our findings confirm other studies conducted in Tanzania [31,32], but are different from data among primary schoolchildren in holoendemic areas of Cameroon [33].

This difference could be attributed to low malaria prevalence in the study area or high mosquito net coverage which was not assessed in the present study. A particular concern is the high occurrence of parasitic co-infections, with concomitant infections of *S. mansoni *and hookworms being the most common. Co-infections of helminth and *P. falciparum *infections also have clinical importance [34]. Previous studies have clearly documented the relationship between intestinal helminth infections, polyparasitism and cognitive functions, growth and malnutrition among school children [35,36]. Hookworm anemia may be exacerbated by infection with other parasites [29]. Children with multiple parasitic infections especially those with heavy infections intensity tend to experience more severe cognitive outcomes and other health problems such as malnutrition than children with only one helminth infection [36,37]. Studies suggest that co-infections of *P. falciparum *with hookworms or schistosomes tend to exacerbate hepato-splenic, anaemia and malnutrition morbidities among school children [38].

Co-infections of *S. mansoni *and hookworm could partly be attributed to the co-endemicity of the two species in the study area and poor sanitations [28,39,40]. This observation is similar to previously reports from Brazil [28] Côte d'Ivoire [27] and China [40]. The polyparasitism of *S. mansoni *and *P. falciparum *observed in the present study have also been observed previously from Senegal [41] and Zimbabwe [42] where prevalence of *P. falciparum *among schoolchildren were high in those infected with *S. mansoni*. One reason for this observation could be the availability of breeding sites for the intermediate host (fresh water snail) and malaria vectors (*Anopheles *mosquitoes) in the study area. Furthermore, heavy intensities of *S. mansoni *infection did not correlate with having heavy intensities of *P. falciparum*. The findings of our study were similar to a study from Uganda [38].

Hookworm and *P. falciparum *co-infections were more common in our study than reports from Cameroon (0.2%) [33] but lower than in Zimbabwe (12.6%) and Western Kenya (41.5%) [25,43]. Difference in geographical variations in exposure and endemicity could be one of the reasons for these differences. However, in this study, *P. falciparum *infection showed no significant association with hookworms.

Similar to our results, triple infections of *P. falciparum*, hookworm and *S. mansoni *have been reported in studies from Côte d'Ivore, Cameroon and Zimbabwe [39,33,42]. These studies conducted throughout Africa and China indicate clearly that most parasitic infections do not occur singly but as co-infections [28,29,39,40]. Although both malaria and helminths have distinct means of transmission patterns, a variety of environmental and host factors may influence their epidemiological and geographical patterns of infections and diseases [34]. In conclusion, this study revealed that *S. mansoni*, hookworm and *P. falciparum *are prevalent among schoolchildren in the study area. The findings of the present study, supports the need for initiatives to implement a new framework for an integrated approach in disease management. We also recommend longitudinal studies to identify the associations between parasites and associated morbidity.

## Competing interests

The authors declare that they have no competing interests.

## Authors' contributions

HDM, GMM, RW and NJSL designed the study and participated in data collection. JS and AK assisted in data collection. JH, HDM, SEM, LML, AMM and EJK analysed the data. HDM and SEM wrote the first draft of the manuscript. All authors contributed to the manuscript and approved its final version.
